# Fracture of the Skull—with Compression of the Brain, Successfully Treated

**Published:** 1838-02-28

**Authors:** S. Stones

**Affiliations:** Philadelphia


*Fracture of the Skull—with Compression of the Brain, successfully treated.* By S. Stones, M. D., of Philadelphia.

I was called to see a coloured boy 18 months old, who had been struck on the head a little above the right ear by an oyster shell. I requested my friend Dr. J. K. Mitchell, to visit the patient with me. On examining the wound, a portion of the shell was found to be very firmly wedged in the bone, producing pressure on the brain, indicated by the usual symptoms.

A portion of the bone, including the shell, was removed by Dr. Mitchell by the Trephine; the dura mater was perfectly sound, very little blood was lost before or during the operation. Towards the close of the operation, a violent convulsion occurred, succeeded by another after an interval of about twenty minutes. The wound was dressed with adhesive strips, and a soft poultice covered the whole, an injection of Lac Assaf. was also directed.

There was no secondary hemorrhage. The patient continued doing well, and in three weeks the wound was entirely healed.

The result of this case is remarkable, when we take into consideration the numerous obstacles tending to retard its progress. Living in a filthy and impoverished home, deprived by day of parental care, the difficulty of keeping the dressings on the wound; (the child tearing them off almost as soon as applied) are alone circumstances calculated to discourage the faintest hopes of a favourable result.

The patient has a vigorous constitution, and has been, almost from its birth, accustomed to hard usage; the greatest part of the day running about the street, exposed to the weather, barefoot, and with scarcely clothing sufficient to conceal nakedness.

To these latter circumstances, may, I think, be mainly attributed the success of the case.

